# Immediate Restoration of Single-Piece Zirconia Implants: A Prospective Case Series—Long-Term Results after 11 Years of Clinical Function

**DOI:** 10.3390/ma14226738

**Published:** 2021-11-09

**Authors:** Elisabeth Steyer, Valentin Herber, Martin Koller, Dániel Végh, Khaled Mukaddam, Norbert Jakse, Michael Payer

**Affiliations:** 1 Department of Dentistry and Oral Health, Division of Restorative Dentistry, Periodontology and Prosthodontics, Medical University of Graz, Billrothgasse 4, 8010 Graz, Austria; elisabeth.amberger@medunigraz.at (E.S.); martin.koller@medunigraz.at (M.K.); 2 Department of Dentistry and Oral Health, Division of Oral Surgery and Orthodontics, Medical University of Graz, Billrothgasse 4, 8010 Graz, Austria; vegh.daniel@dent.semmelweis-univ.hu (D.V.); norbert.jakse@medunigraz.at (N.J.); mi.payer@medunigraz.at (M.P.); 3 Department of Orthopedics and Traumatology, Medical University of Graz, Auenbruggerplatz 5/6, 8036 Graz, Austria; 4 Department of Prosthodontics, Semmelweis University, Szentkiralyi utca 47, 1088 Budapest, Hungary; 5 University Center for Dental Medicine Basel, Department of Oral Surgery, University of Basel, Mattenstrasse 40, 4058 Basel, Switzerland; khaled.mukaddam@unibas.ch

**Keywords:** CAD/CAM, immediate loading, radiographic bone level, zirconia implants

## Abstract

Objectives: The aim of this prospective case series was to evaluate single-piece zirconia implants restored with lithium disilicate CAD/CAM crowns through a long-term follow-up. Methods: In this trial, 20 one-piece zirconia implants were placed in 20 patients. Implants were restored (i) immediately with lithium disilicate CAD/CAM provisionals, and (ii) permanently four months after surgery. Patients were followed for 11 years. Clinical parameters and radiological measurements of the zirconia implants were assessed. For the statistical analysis, paired *t*-test was applied. Results: Four implants were counted as implant failure due to the loss of implant stability, resulting in a Kaplan–Meier survival rate of 80% up to 11 years. The mean bleeding on probing values were 19.1% (SD ± 13.1) and 18.2% (SD ± 17.6) 96 and 11 years after implant placement, respectively. The plaque index revealed a significant decrease over time (*p* < 0.001) with a value between 25.9% (SD ± 5.7) and 12.6% (SD ± 10.0) at baseline and 11-years follow-up respectively. The marginal bone level revealed a significant decrease 4, 8, and 11 years after implant insertion (*p* = 0.001, *p* = 0.019, and *p* = 0.027, respectively). Conclusions: Immediately loaded zirconia single-piece implants showed a suitable success rate in clinical and radiographic outcomes.

## 1. Introduction

Since the 1980s and the introduction of dental implant therapy by Per-Ingvar Brånemark, the replacement of missing teeth using implant-supported restorations is a daily and globally-accepted clinical approach [1]. Titanium and its alloys are commonly used in implant dentistry and various clinical studies have demonstrated their high survival rates [2]. However, several concerns have been expressed with regard to the titanium particles and its implications in inflammatory reactions in peri-implant tissues [3,4]. When compared, Safioti et al. reported the presence of dissolved titanium at higher quantities in the submucosal biofilm taken from implants with peri-implantitis, versus biofilm taken from healthy implants [5]. The local concentration of titanium particles, mainly distance-dependent, may result in damage to the intraepithelial hemostasis, bone resorption through a stimulation of osteoclast differentiation, and implant detachment [6].

Subsequently, a heightened demand for metal-free implants has amplified the quest for alternative materials over the last few years. Zirconia ceramic implants made with yttria-stabilized tetragonal zirconia polycrystal (Y-TZP) have been widely investigated. Patients with thin gingival biotype and the need for implant placement in the aesthetic area might benefit from the tooth-like color of zirconia [7]. Moreover, osseointegration of zirconia implants has been preclinically shown to be vital for implant success and related survival rates [8]. Apposition of bone may also be influenced by the implant surface topography modified through sandblasting, etching, sintering, or coating [8,9,10]. Thus, hard tissue integration around zirconia implants can be optimized, allowing for increased implant success. Regarding biofilm formation on zirconia implants, rather controversial results can be found. Kniha et al. recently presented in a meta-analysis that the bacterial coverage of zirconia surfaces was significantly superior than titanium surfaces [11]. Other in vitro studies have exhibited significant reduction in human biofilm formation on zirconia implant surfaces [11,12]. Nevertheless, numerous short- and mid-term clinical studies have shown excellent biocompatibility and promising outcomes of zirconia implants [13,14]. Even in experimental mucositis conditions, clinical evaluation of zirconia implants showed lower plaque and bleeding scores compared to titanium [15].

Conversely, some drawbacks of zirconia such as aging and degradation at low temperatures can be found in the current literature. Compressive stresses and microcracking can lead to material aging [16].

Soft and hard tissue integration has to be investigated clinically and only a few clinical studies have reported long-term outcomes [17]. Lorusso et al. recently reported only 29 clinical studies investigating zirconia implants, where 26 of them had been published during the last ten years and most (21) originated from Europe, particularly in German-speaking countries [18].

Single-piece zirconia implants have mostly been documented. The design of these single-piece implants permit the avoidance of microgaps, which can be associated with reduced marginal bone loss by decreasing the accumulation of biofilm [19]. Single-piece zirconia implants can be ground intraorally as zirconia has a low thermal conductivity. Bethke et al. pointed out that single-piece implants showed higher fracture resistance compared to two-piece zirconia implants in vitro, but lower bending moments after grinding [20] in contrast to former findings [21]. Controversially, single-piece implants supporting lithium disilicate crowns demonstrated promising mid-term clinical outcomes [22,23].

Based on these aspects, the study aims to report the clinical and radiographical outcomes of lithium disilicate and microgap-less crowns on zirconia single-piece implants four, eight, and 11 years after implant placement, updating previously reported data that covered 24 months [24].

## 2. Materials and Methods

### 2.1. Study Design and Population

Between the years 2008 and 2009, a total of 20 healthy patients were included in this single center prospective case series. All participants had to fulfil the defined inclusion criteria: patients of 18 years of age or older who had given their informed, written consent; with single-tooth gaps without vertical or horizontal bony defects and characterized by a sufficient soft-tissue volume for the placement of implants with a minimum length of 10 mm and 3.5 mm width. Additionally, the participants had to accept the scheduled protocol of clinical and radiographic analysis and maintenance. The exclusion criteria were smokers with more than nine cigarettes per day; homelessness; signs of occlusal parafunctions (e.g., bruxers); patients with lack of compliance or failure to provide their informed consent; presence of acute periodontal disease; general contraindications against implant treatment, metabolic skeletal disease (e.g., osteomalacia or osteoporosis), or medication potentially affecting bone structure and metabolism (corticosteroid treatment or bisphosphonate or denosumab therapy); pregnancy assessed with a pregnancy test; previous irradiation in the neck/head area; need for bone augmentation procedures; or need for the replacement of more than one neighboring tooth.

This clinical trial was performed in the Department of Dentistry and Oral Health at the Medical University of Graz, approved by the local ethics committee (19/204 ex 07/08) and conducted according to the Good Clinical Practice (ISO 14155:2011) standard and the Declaration of Helsinki.

### 2.2. Clinical Procedures

Between the years 2008 and 2009, 20 single-piece zirconia implants (whiteSKY^®^, Bredent medical, Senden, Germany) were placed in 20 patients by two experienced surgeons. These single-piece zirconia implants were made with an yttrium oxide (Y_2_O_3_)-stabilized tetragonal polycrystalline zirconium oxide. The surface roughness was achieved through sandblasting [25]. Five implants were inserted in the upper incisor, six in the maxillary premolar region, eight in the lower molar, and one implant in the mandibular premolar region.

After placement, the implants were immediately ground intraorally without touching the implant shoulder and digital impressions were performed using a CAD/CAM system (Cerec 3D^®^, DentsplySirona, Bensheim, Germany). Provisional crowns made with monolithic lithium disilicate (e.max^®^ CAD-Blocks, Ivoclar-Vivadent, Schaan, Liechtenstein) were then fabricated according to the digital impression using the Cerec 3D^®^ system (with powdering) (Cerec 3D^®^, DentsplySirona, Bensheim, Germany). Lithium disilicate provisional single crowns were constructed without centric or eccentric occlusal contacts for four months to avoid functional loading and guarantee a stress-free healing period. The temporary crowns were luted adhesively (Multilink-Automix^®^, Ivoclar-Vivadent, Schaan, Liechtenstein) using a rubber dam to reach a microgap-less implant–crown connection.

After a healing time of four months, the implants were subsequently restored with permanent lithium disilicate lithium disilicate crowns (e.max^®^ CAD-Blocks, Ivoclar-Vivadent, Schaan, Liechtenstein). To manufacture the permanent crown, the provisional crowns were ground and served as lithium disilicate caps for the final restoration. Conventional impressions were taken with polyether (Impregum NF^®^, 3M ESPE, Seefeld, Germany) and permanent lithium disilicate lithium disilicate crowns were manufactured. Individual crowns were then adhesively (Multilink-Automix^®^, Ivoclar-Vivadent, Schaan, Liechtenstein) luted using the same procedure as for the provisional one.

### 2.3. Clinical Evaluation

For each patient, the study consisted of clinical assessments conducted at baseline (after crown placement) and 4, 8 and 11 years after implant placement. Bleeding on probing (BOP) [3] present or non-present around the teeth and implant, respectively, plaque index (PI) [26], and implant stability (Periotest^®^; PTV; Medizintechnik Gulden e. K., Modautal, Germany) were evaluated. Several intraoral photographs (D80, Nikon^®^, Tokyo, Japan) of the implant prosthetics and the surrounding tissues were taken at baseline and each visit. The success of an implant was defined as an implant in situ supporting a prosthetic superstructure, showing no periotest values ≥8, no implant induced-pain or peri-implant translucency, no infection or paraesthesia, no implant fracture, and no peri-implant marginal bone loss of more than 1.5 mm during the first year in function and no annual bone loss exceeding 0.2 mm in the following years, according to Naert et al. [27], Snauwaert et al. [28] and Buch et al. [29].

### 2.4. Radiographic Evaluation

Standardized single tooth radiographs (Sidexis^®^ Intraoral, Orthophos plus DS; DentsplySirona, Bensheim, Germany) were taken just after crown placement (baseline) and 4, 8, and 11 years after implant placement with a rectangular collimation technique using a Sirona XG AimRight sensor holder system (DentsplySirona, Bensheim, Germany). The marginal bone level (MBL) was assessed by one investigator (E.S.). Implant dimension (diameter) served as a reference for the calibration and evaluation of the related parameter (MBL). The reference point for the measurement of the mesial and distal bone contour was the implant shoulder. The assessment of MBL was expressed as mean ± standard deviation (SD). If the lingual and buccal bone contours overlapped, a mean value was chosen. In questionable cases, a consensus of two investigators was considered.

### 2.5. Statistical Analysis

A descriptive statistical analysis of all radiographic and clinical parameters (BOP, PI, and MBL) abovementioned was performed. The data (BOP, PI, MBL) were statistically evaluated at several timepoints (baseline, four, eight, and 11 years) after implant placement using the paired *t*-test. *p*-values below 0.05 were considered to be significant. Kaplan–Meier survival rate were additionally calculated. The data were analyzed using SPSS^®^ software version 25 (SPSS, Chicago, IL, USA) and SAS^®^ software version 9.4 (SAS Institute, Cary, NC, USA).

## 3. Results

### 3.1. Patient Information

The 20 participants, divided into eight females (40%) and 12 males (60%), showed a mean age of 42.9 years (SD ± 12.7) and 43.7 years (SD ± 16.1), respectively. Overall, the average age was 43.3 years (SD ± 14.5) (Table 1).

Eleven of the evaluated implants (55%) were located in the maxilla and nine in the lower jaw (45%). Eight (40%) single-piece zirconia implants were placed in the first lower molar region (Table 2).

Implant dimensions are shown in Table 3.

A total of 16 subjects of the 20 patients initially included in the trial remained for the follow-up examinations. One early implant loss, four months after implantation, was already reported in the first publication [24]. Three other dental implants were lost in the period between 24- and 48-months after implant placement (Table 4).

**Figure 1:**
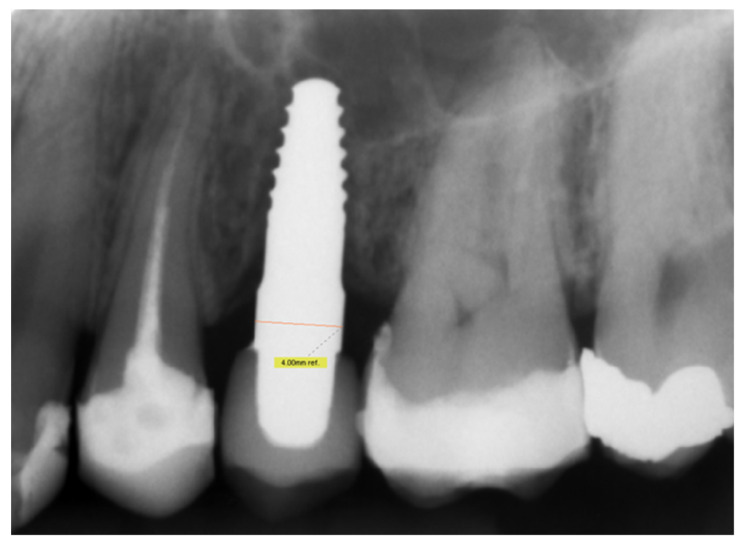
Single tooth radiograph of a 34-year-old male patient 25 months after implantation. The bone loss at the implant site was clearly visible, while the crestal bone level of the adjacent teeth was not affected.

Initially, peri-implantitis was designated as the main reason for failure in all three cases. Two of these three implants of 3.5 mm width and 10 mm length, respectively, expressed an increasing loss of implant stability and corresponding increase in mobility and bone loss without general signs of peri-implantitis. The crestal bone level of the adjacent teeth also showed no reduction (Figure 1). Additionally, the third implant loss was caused by peri-implantitis. No implant fracture was reported. The related four participants were declared as failures.

Five additional patients were lost to follow-up for various reasons. Of these five patients, one participant died 57 months after implant placement with the implant in situ. Another participant, who suffered of severe illnesses eight years after implant placement, could not be reached for further examination. Another three patients were lost to follow-up due to loss of contact (two participants after the 24-month follow-up, one after the 48-month follow-up). One patient denied undergoing the periodontal examination at the 8- and 11-years follow-up. In total, 11 patients in this case series could be observed radiographically over a period of 11 years.

### 3.2. Clinical Assessments

**Figure 2:**
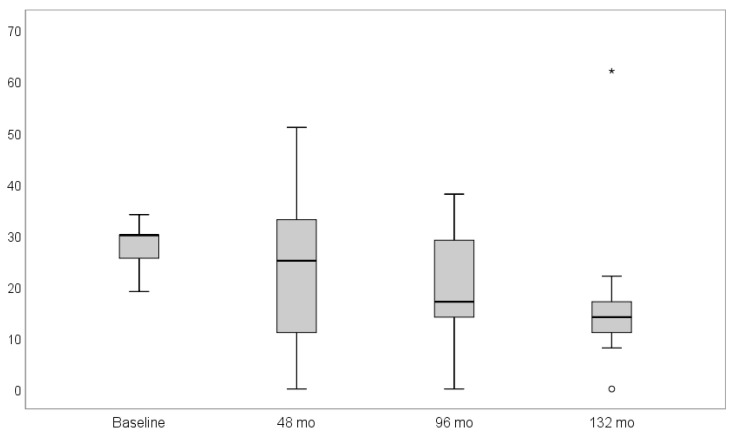
Box-plot diagram of bleeding on probing (BOP) scores at baseline, four, eight, and 11 years after follow-up. The bleeding on probing revealed no significant decrease over time (*p* = 0.165). Symbols (star and circle) indicate outliers of the study sample.

The mean BOP values at follow-up were 27.9% (SD ± 4.2), 23.8% (SD ± 15.6), 19.1% (SD ± 13.1), and 18.2% (SD ± 17.6) at baseline, four, eight, and 11 years after implant placement, respectively. At the 11-year follow-up, two statistical outliers were discovered. One participant showed no BOP while another gained a BOP of 62% (Figure 2). However, no statistical significance on BOP values could be assessed over time (*p* = 0.165).

**Figure 3:**
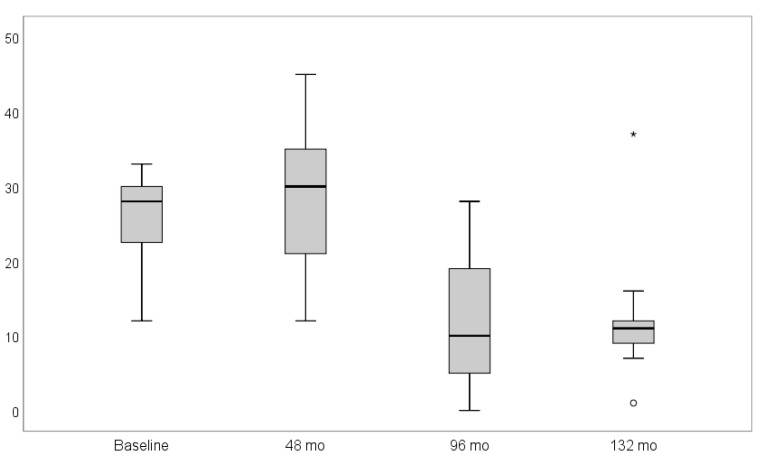
Box-plot diagram of the plaque index (PI) scores at baseline, four, eight, and 11 years after follow-up. The plaque index revealed a significant decrease over time (*p* < 0.001). Symbols (star and circle) indicate outliers of the study sample.

The PI values revealed a mean of 25.9% (SD ± 5.7), 28.5% (SD ± 11.0), 12.6% (SD ± 9.4), and 12.6% (SD ± 10.0) at the baseline and 4-, 8-, and 11-year follow-ups respectively. Eleven years after implant insertion, two statistical outliers (1% vs. 37%) concerning the PI were detected (Figure 3). A statistically significant decrease in PI over time, according to the paired t-test, could be noticed (*p* < 0.001). All results are presented in Table 5.

**Figure 4:**
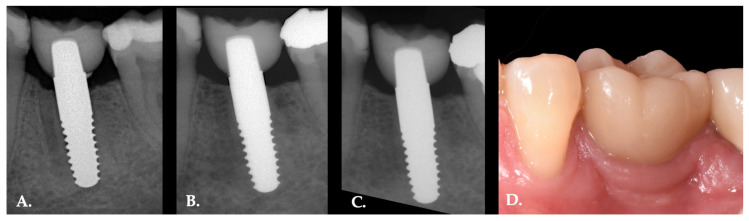
Intraoral radiographs of a single piece zirconia implant placed in 36 in a 39-year-old female patient at baseline (**A**), 4 years (**B**) and 11 years (**C**) after implant placement. Intraoral photograph of the definitive restauration 11 years (**D**) after implantation.

All remaining implants up to 11 years could be stated as stable and successfully integrated (Figure 4). The calculated Kaplan–Meier success rate was 80% [30]. No implant fracture or prosthetic complications such as loss of implant superstructure or chipping was observed after the observation period of 11 years. Patients reported no implant induced pain or paraesthesia.

### 3.3. Radiographic Assessments

**Figure 5:**
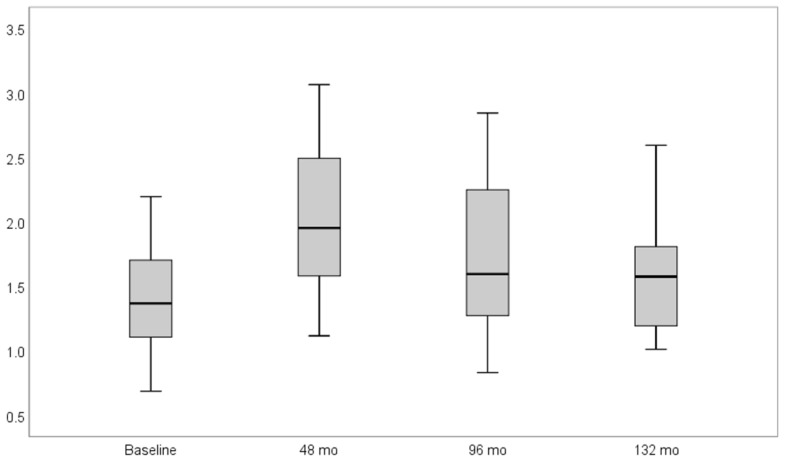
Box-plot diagram of marginal bone level (MBL) scores at baseline, four, eight, and 11 years after implant placement. In relation to the baseline, the marginal bone level revealed a significant decrease four, eight, and 11 years after implant insertion (*p* = 0.001, *p* = 0.019, and *p* = 0.027, respectively).

From the remaining 16 implants included in this long-term follow-up case series, 11 could be observed over the whole period of 11 years concerning the MBL parameter. At baseline, 4-, 8-, and 11-year follow-ups, a mean MBL of 1.4 mm (SD ± 0.43), 1.99 mm (SD ± 0.57), 1.70 mm (SD ± 0.64), and 1.59 mm (SD ± 0.53), respectively, were reported. Four years after implant placement, a statistically significant resorption of the marginal bone over time was detected (*p* = 0.005). Even if a little less clearly, a statistical significance in terms of MBL was also shown after eight (*p* = 0.019) and 11 years (*p* = 0.027) post implant placement (Figure 5). The bone loss with reference to the baseline MBL did not exceed −1.27 mm after 11 years of follow-up. All results are presented in Table 5.

## 4. Discussion

This case series, initially including 20 patients recruited between 2008 and 2009, provides long-term clinical and radiographic outcomes of 11 immediately restored zirconia single-piece implants up to 11 years of clinical functions. To the authors’ knowledge, this is the only clinical trial reporting zirconia implants clinical data up to 11 years.

In any study involving long-term follow-up, higher rate of losses to follow-up are expected primarily due to patient relocation [31]. In this case-series, four participants were lost to follow-up. These patients were contacted, and they confirmed that the single piece zirconica implants were in situ, but the patients did not send the radiographs and were not able to visit the clinical center for further investigations.

Concerning implant survival, Payer et al. reported one implant failure four months after provisional restoration [24]. Three other implant failures placed in the upper jaw occurred within the first 48 months after implant placement. A recent systematic review exhibited a general influence of implant location on the estimated implant loss rate with a significantly higher implant loss in the maxilla [32].

Additionally, Hashim et al. reviewed that the one-piece zirconia implant exhibited a general tendency for early implant loss [17]. In this study, zirconia implants were immediately restored with lithium disilicate CAD/CAM provisionals. The provisional crowns did not have any centric or eccentric occlusal contacts to avoid functional loading and guarantee a stress-free healing period. However, implants are still exposed to indirect forces during the masticatory process and tongue movements.

Implant osseointegration, and therefore implant survival rate, is also influenced by the surface topography. The used single-piece zirconia implant showed an average surface roughness (Sa) of 1.17 µm (±0.15 µm) [33], which can be considered as a moderately rough surface (Sa values between 1.1–2 µm) [34]. Titanium implants with minor surface roughness are more likely to fail [35]. Hard tissue peri-implant integration is also dependent on the cleanness of the surface. Impurities may derive from the manufacturing handling or packaging processes [36]. In our cases, this explanation is not plausible as these implants were previously documented and their sterile packages were periodically reviewed.

Furthermore, the four reported implant failures had a diameter of 3.5 mm (Table 4). Olate et al. reported, through a retrospective study including 1649 implants, that the largest implant loss was observed in narrow implants [37]. However, this information cannot be generalized as a recent systematic review and meta-analysis showed no statistical significant differences in implant survival between narrow implants with a diameter between 3.30–3.50 mm compared to standard implant (diameter >3.5 mm) [38].

The reported implant failures did not show any general inflammatory signs; thus some authors have explained these cases by an “aseptic loosening” [7]. Disintegration or premature loading were quoted in the literature as a possible reason [14].

In the present study, the appearance of plaque at zirconia implants revealed a significant decrease over time (*p* < 0.001). The former thesis that explains the lower accumulation of biofilm around the zirconia implant may explain this clinical outcome [15]. Additionally, from a periodontal perspective, a minor surface roughness may explain a lower bacterial adhesion in comparison to major surface roughness [39]. Similar promising results for BOP and PI were also recently published in a mid-term report with up to 80 months of follow-up of two-piece zirconia implants [40].

Regarding the radiographic evaluation, the marginal bone level revealed a significant decrease 48 months after implant insertion (*p* = 0.001), while this level was stable at the 96- and 11-year follow-ups with a mean value 0.2 mm lower than the initial measure. Albrektsson et al. reported a higher amount of bone loss during the first year of function, followed by a stable bony situation after osseointegration [41]. High marginal bone loss was also exhibited during the first 8–16 weeks after implant insertion even before the prosthetic approach [22]. Mid-term results of this trial showed a stable bone level. This preservation may be inextricably linked to the high compatibility, less plaque adhesion, and the microgap-less implant design of single-piece zirconia implants [42].

The reported one-piece zirconia implant system was launched into the market in 2006 and is still commercially available. A slightly modified version (e.g., two different neck designs and optimized abutment height to minimize the need of intraoral individualization and grinding, respectively) was released this year (Bredent Medical GmbH, Germany).

In the present study, immediately loaded single-piece zirconia implants showed overall clinical and functional stable outcomes despite the report of early implant failures. As stated in the literature, an essential requirement for successful immediate loading of dental implants is an optimal primary implant stability (>32 N·cm^−1^) [43]. Additionally, the success of immediately loaded implants is inextricably linked to careful patient selection associated with prosthetic factors [44]. A load protection of the provisional restauration without centric or eccentric occlusal contacts that guarantee a stress-free healing period might influence the crestal bone level, and therefore the success of the implant [45,46,47]. Additionally, the immediate provisional restorations of this two-step technique can form a desirable emergence profile that can be transferred accurately to the final restoration. The contribution of immediate provisional restorations associated with the maintenance of peri-implant health is inextricably linked to the reduction in the risk of peri-implant disease associated with excess cement [48].

In the present immediate protocol, CAD/CAM lithium disilicate crowns were used. The material used for the provisional and final restorations needs to be considered as the CAD/CAM lithium disilicate ceramic in a monolithic configuration showed a fatigue-resistant property in comparison with the veneered zirconia [49,50].

Therefore, a combination of patient, surgical, and prosthetic parameters must be considered for immediate loading protocols.

A major drawback of the present study to be considered is the missing control group. Moreover, the reliability of the results should be interpreted with caution due to the low number of participants included in the case series. Furthermore, only data extracted from a randomized clinical trial with a long-term outcome can allow for a final elucidation of zirconia as an alternative implant material.

## 5. Conclusions

The present case series of 20 immediately restored zirconia single-piece implants presented four early implant failures, five losses to follow-up, and stable radiographic and clinical outcomes of the remaining 11 implants after a follow-up of up to 11 years and the related problems arising from loss to follow-up. Immediately loaded zirconia single-piece implants showed a suitable success rate, clinical, and radiographic outcomes. However, a larger prospective randomized controlled clinical trial needs to be performed to confirm the validity of these results.

## Figures and Tables

**Table 1:** Demographic data.

Gender/Age	N	%	Age Min	Age Max	Age Mean	SD
Female	8	40	31	70	42.9	12.7
Male	12	60	25	69	43.7	16.1
Total	20	100	26	70	43.3	14.5

**Table 2:** Implant location.

Implant Site	N	%
11	1	5
12	1	5
13	1	5
14	2	10
21	2	10
24	2	10
25	2	10
35	1	5
36	3	15
46	5	25

**Table 3:** Distribution of the placed implants (n) including implant length (mm) and diameter (mm).

		Implant Diameter (mm)		
		3.5	4	4.5
Implant length (mm)	10	2	-	-
	12	4	6	3
	14	2	2	1

**Table 4:** Distribution of the lost implants (n) including details about implant length (mm) and diameter (mm).

		Implant Diameter (mm)		
		3.5	4	4.5
Implant length (mm)	10	2	-	-
	12	1	-	-
	14	1	-	-

**Table 5:** Clinical (BOP and PI) and radiographic (MBL) parameters evaluated at baseline, 4-, 8-, and 11-years after implant placement.

		N	Min	Max	Mean	SD	*p* Value
BOP	baseline	16	19	34	27.9	4.0	
	4 years	13	0	51.0	23.8	15.6	*p* > 0.05
	8 years	10	0	38	19.1	13.1	*p* > 0.05
	11 years	9	0	62	18.2	17.6	*p* = 0.165
PI	baseline	16	12	33	25.3	5.7	
	4 years	13	12	45	28.5	11.0	*p* > 0.05
	8 years	10	0	28	12.6	9.4	*p* > 0.05
	11 years	9	1	37	12.6	10.0	*p* < 0.001
MBL	baseline	16	0.69	2.19	1.40	0.43	
	4 years	14	1.12	3.07	1.99	0.57	*p* = 0.001
	8 years	12	0.83	2.85	1.70	0.64	*p* = 0.019
	11 years	11	1.01	2.60	1.59	0.53	*p* = 0.027
	resorption	14	−0.55	1.27	0.43	0.48	*p* = 0.005

## Data Availability

The data presented in this study are available on request from the corresponding author. The data are not publicly available due to ethical reasons.

## References

[1] Bassir S.H., El Kholy K., Chen C.-Y., Lee K.H., Intini G. (2019). Outcome of Early Dental Implant Placement versus Other Dental Implant Placement Protocols: A Systematic Review and Meta-Analysis. J. Periodontol..

[2] Buser D., Sennerby L., De Bruyn H. (2017). Modern Implant Dentistry Based on Osseointegration: 50 Years of Progress, Current Trends and Open Questions. Periodontology 2000.

[3] Mombelli A., van Oosten M.A., Schurch E., Land N.P. (1987). The Microbiota Associated with Successful or Failing Osseointegrated Titanium Implants. Oral Microbiol. Immunol..

[4] Fretwurst T., Nelson K., Tarnow D.P., Wang H.-L., Giannobile W.V. (2018). Is Metal Particle Release Associated with Peri-Implant Bone Destruction? An Emerging Concept. J. Dent. Res..

[5] Safioti L.M., Kotsakis G.A., Pozhitkov A.E., Chung W.O., Daubert D.M. (2017). Increased Levels of Dissolved Titanium Are Associated With Peri-Implantitis—A Cross-Sectional Study. J. Periodontol..

[6] Zhou Z., Shi Q., Wang J., Chen X., Hao Y., Zhang Y., Wang X. (2021). The Unfavorable Role of Titanium Particles Released from Dental Implants. Nanotheranostics.

[7] Cionca N., Hashim D., Mombelli A. (2017). Zirconia Dental Implants: Where Are We Now, and Where Are We Heading?. Periodontology 2000.

[8] Bosshardt D.D., Chappuis V., Buser D. (2017). Osseointegration of Titanium, Titanium Alloy and Zirconia Dental Implants: Current Knowledge and Open Questions. Periodontology 2000.

[9] Schünemann F.H., Galárraga-Vinueza M.E., Magini R., Fredel M., Silva F., Souza J.C.M., Zhang Y., Henriques B. (2019). Zirconia Surface Modifications for Implant Dentistry. Mater. Sci. Eng. C Mater. Biol. Appl..

[10] Hafezeqoran A., Koodaryan R. (2017). Effect of Zirconia Dental Implant Surfaces on Bone Integration: A Systematic Review and Meta-Analysis. BioMed Res. Int..

[11] Kniha K., Heussen N., Modabber A., Hölzle F., Möhlhenrich S.C. (2021). The Effect of Zirconia and Titanium Surfaces on Biofilm Formation and on Host-Derived Immunological Parameters. Int. J. Oral Maxillofac. Surg..

[12] Al-Radha A.S.D., Dymock D., Younes C., O’Sullivan D. (2012). Surface Properties of Titanium and Zirconia Dental Implant Materials and Their Effect on Bacterial Adhesion. J. Dent..

[13] Haro Adánez M., Nishihara H., Att W. (2018). A Systematic Review and Meta-Analysis on the Clinical Outcome of Zirconia Implant-Restoration Complex. J. Prosthodont. Res..

[14] Roehling S., Schlegel K.A., Woelfler H., Gahlert M. (2018). Performance and Outcome of Zirconia Dental Implants in Clinical Studies: A Meta-Analysis. Clin. Oral Implants Res..

[15] Bienz S.P., Hilbe M., Hüsler J., Thoma D.S., Hämmerle C.H.F., Jung R.E. (2021). Clinical and Histological Comparison of the Soft Tissue Morphology between Zirconia and Titanium Dental Implants under Healthy and Experimental Mucositis Conditions-A Randomized Controlled Clinical Trial. J. Clin. Periodontol..

[16] Lughi V., Sergo V. (2010). Low Temperature Degradation-Aging-of Zirconia: A Critical Review of the Relevant Aspects in Dentistry. Dent. Mater..

[17] Hashim D., Cionca N., Courvoisier D.S., Mombelli A. (2016). A Systematic Review of the Clinical Survival of Zirconia Implants. Clin. Oral Investig..

[18] Lorusso F., Noumbissi S., Francesco I., Rapone B., Khater A.G.A., Scarano A. (2020). Scientific Trends in Clinical Research on Zirconia Dental Implants: A Bibliometric Review. Materials.

[19] Borgonovo A.E., Censi R., Vavassori V., Dolci M., Calvo-Guirado J.L., Delgado Ruiz R.A., Maiorana C. (2013). Evaluation of the Success Criteria for Zirconia Dental Implants: A Four-Year Clinical and Radiological Study. Int. J. Dent..

[20] Bethke A., Pieralli S., Kohal R.-J., Burkhardt F., von Stein-Lausnitz M., Vach K., Spies B.C. (2020). Fracture Resistance of Zirconia Oral Implants In Vitro: A Systematic Review and Meta-Analysis. Materials.

[21] Silva N.R.F.A., Coelho P.G., Fernandes C.A.O., Navarro J.M., Dias R.A., Thompson V.P. (2009). Reliability of One-Piece Ceramic Implant. J. Biomed. Mater. Res. B Appl. Biomater..

[22] Kohal R.-J., Spies B.C., Vach K., Balmer M., Pieralli S. (2020). A Prospective Clinical Cohort Investigation on Zirconia Implants: 5-Year Results. J. Clin. Med..

[23] Balmer M., Spies B.C., Kohal R.-J., Hämmerle C.H.-F., Vach K., Jung R.E. (2020). Zirconia Implants Restored with Single Crowns or Fixed Dental Prostheses: 5-Year Results of a Prospective Cohort Investigation. Clin. Oral Implants Res..

[24] Payer M., Arnetzl V., Kirmeier R., Koller M., Arnetzl G., Jakse N. (2013). Immediate Provisional Restoration of Single-Piece Zirconia Implants: A Prospective Case Series—Results after 24 Months of Clinical Function. Clin. Oral Implants Res..

[25] Beger B., Goetz H., Morlock M., Schiegnitz E., Al-Nawas B. (2018). In Vitro Surface Characteristics and Impurity Analysis of Five Different Commercially Available Dental Zirconia Implants. Int. J. Implant Dent..

[26] O’Leary T.J., Drake R.B., Naylor J.E. (1972). The Plaque Control Record. J. Periodontol..

[27] Naert I., Quirynen M., van Steenberghe D., Darius P. (1992). A Six-Year Prosthodontic Study of 509 Consecutively Inserted Implants for the Treatment of Partial Edentulism. J. Prosthet. Dent..

[28] Snauwaert K., Duyck J., van Steenberghe D., Quirynen M., Naert I. (2000). Time Dependent Failure Rate and Marginal Bone Loss of Implant Supported Prostheses: A 15-Year Follow-up Study. Clin. Oral Investig..

[29] Buch R.S.R., Weibrich G., Wagner W. (2003). Criteria of success in implantology. Mund-Kiefer-Und Gesichtschirurgie MKG.

[30] Kaplan E.L., Meier P. (1958). Nonparametric Estimation from Incomplete Observations. J. Am. Stat. Assoc..

[31] Palta M., McHugh R. (1979). Adjusting for Losses to Follow-up in a Sample Size Determination for Cohort Studies. J. Chronic Dis..

[32] Kern J.-S., Kern T., Wolfart S., Heussen N. (2016). A Systematic Review and Meta-Analysis of Removable and Fixed Implant-Supported Prostheses in Edentulous Jaws: Post-Loading Implant Loss. Clin. Oral Implants Res..

[33] Borgonovo A.E., Arnaboldi O., Censi R., Dolci M., Santoro G. (2010). Edentulous Jaws Rehabilitation with Yttrium-Stabilized Zirconium Dioxide Implants: Two Years Follow-up Experience. Minerva Stomatol.

[34] Zinelis S., Thomas A., Syres K., Silikas N., Eliades G. (2010). Surface Characterization of Zirconia Dental Implants. Dent Mater..

[35] Oh S.-L., Shiau H.J., Reynolds M.A. (2020). Survival of Dental Implants at Sites after Implant Failure: A Systematic Review. J. Prosthet. Dent..

[36] Duddeck D.U., Albrektsson T., Wennerberg A., Larsson C., Beuer F. (2019). On the Cleanliness of Different Oral Implant Systems: A Pilot Study. J. Clin. Med..

[37] Olate S., Lyrio M.C.N., de Moraes M., Mazzonetto R., Moreira R.W.F. (2010). Influence of Diameter and Length of Implant on Early Dental Implant Failure. J. Oral Maxillofac. Surg..

[38] Schiegnitz E., Al-Nawas B. (2018). Narrow-Diameter Implants: A Systematic Review and Meta-Analysis. Clin. Oral Implant. Res..

[39] Quirynen M., Abarca M., Van Assche N., Nevins M., van Steenberghe D. (2007). Impact of Supportive Periodontal Therapy and Implant Surface Roughness on Implant Outcome in Patients with a History of Periodontitis. J. Clin. Periodontol..

[40] Koller M., Steyer E., Theisen K., Stagnell S., Jakse N., Payer M. (2020). Two-Piece Zirconia versus Titanium Implants after 80 Months: Clinical Outcomes from a Prospective Randomized Pilot Trial. Clin. Oral Implants Res..

[41] Albrektsson T., Zarb G., Worthington P., Eriksson A.R. (1986). The Long-Term Efficacy of Currently Used Dental Implants: A Review and Proposed Criteria of Success. Int. J. Oral Maxillofac. Implants.

[42] Borgonovo A.E., Censi R., Vavassori V., Arnaboldi O., Maiorana C., Re D. (2015). Zirconia Implants in Esthetic Areas: 4-Year Follow-Up Evaluation Study. Int. J. Dent..

[43] Esposito M., Grusovin M.G., Maghaireh H., Worthington H.V. (2013). Interventions for Replacing Missing Teeth: Different Times for Loading Dental Implants. Cochrane Database Syst. Rev..

[44] Kan J.Y.K., Rungcharassaeng K., Deflorian M., Weinstein T., Wang H.-L., Testori T. (2018). Immediate Implant Placement and Provisionalization of Maxillary Anterior Single Implants. Periodontology 2000.

[45] Payer M., Heschl A., Wimmer G., Wegscheider W., Kirmeier R., Lorenzoni M. (2010). Immediate Provisional Restoration of Screw-Type Implants in the Posterior Mandible: Results after 5 Years of Clinical Function. Clin. Oral Implant. Res..

[46] Grandi T., Garuti G., Guazzi P., Tarabini L., Forabosco A. (2012). Survival and Success Rates of Immediately and Early Loaded Implants: 12-Month Results from a Multicentric Randomized Clinical Study. J. Oral Implantol..

[47] Ghoul W.E., Chidiac J.J. (2012). Prosthetic Requirements for Immediate Implant Loading: A Review. J. Prosthodont..

[48] Staubli N., Walter C., Schmidt J.C., Weiger R., Zitzmann N.U. (2017). Excess Cement and the Risk of Peri-Implant Disease–A Systematic Review. Clin. Oral Implant. Res..

[49] Kim J.H., Lee S.J., Park J.S., Ryu J.J. (2013). Fracture Load of Monolithic CAD/CAM Lithium Disilicate Ceramic Crowns and Veneered Zirconia Crowns as a Posterior Implant Restoration. Implant. Dent..

[50] Guess P.C., Zavanelli R.A., Silva N.R., Bonfante E.A., Coelho P.G., Thompson V.P. (2010). Monolithic CAD/CAM Lithium Disilicate versus Veneered Y-TZP Crowns: Comparison of Failure Modes and Reliability after Fatigue. Int. J. Prosthodont..

